# Mechanical ventilation patterns and trends over 20 years in an Israeli hospital system: policy ramifications

**DOI:** 10.1186/s13584-019-0291-y

**Published:** 2019-02-01

**Authors:** Rachel Yaffa Zisk-Rony, Charles Weissman, Yoram G. Weiss

**Affiliations:** 1 Hebrew University, Hadassah Henrietta Szold School of Nursing, Jerusalem, Israel; 2 Department of Anesthesiology and Critical Care Medicine, Respiratory Care Service and Hospital Administration, Hadassah-Hebrew University Medical Center, Hebrew University-Hadassah School of Medicine, Kiryat Hadassah POB 12000, 91120 Jerusalem, Israel

**Keywords:** Mechanical ventilation, Seasonality, Internal medicine, Intensive care unit, Respiratory care, Intensive care unit

## Abstract

**Background:**

Mechanical ventilation is a life supporting modality increasingly utilized when caring for severely ill patients. Its increasing use has extended the survival of the critically ill leading to increasing healthcare expenditures. We examined changes in the hospital-wide use of mechanical ventilation over 20 years (1997–2016) in two Israeli hospitals to determine whether there were specific patterns (e.g. seasonality, weekday vs. weekend) and trends (e.g. increases or decreases) among various hospital departments and units.

**Methods:**

Retrospective analysis of prospectively collected data on all mechanically ventilated patients over 20-years in a two-hospital Israeli medical system was performed. Data were collected for each hospital unit caring for ventilated patients. Time-series analysis examined short and long-term trends, seasonality and intra-week variation.

**Results:**

Over two decades overall ventilator-days increased from 11,164 (31 patients/day) in 1997 to 24,317 (67 patients/day) in 2016 mainly due to more patients ventilated on internal medicine wards (1997: 4 patients/day; 2016: 24 patients/day). The increases in other hospital areas did not approach the magnitude of the internal medicine wards increases. Ventilation on wards reflected the insufficient number of ICU beds in Israel. A detailed snapshot over 4 months of patients ventilated on internal medicine wards (*n* = 745) showed that they tended to be elderly (median age 75 years) and that 24% were ventilated for more than a week. Hospital-wide ventilation patterns were the weighted sum of the various individual patient units with the most noticeable pattern being peak winter prevalence on the internal medical wards and in the emergency department. This seasonality is not surprising, given the greater incidence of respiratory ailments in winter.

**Conclusions:**

Increased mechanical ventilation plus seasonality have budgetary, operational and staffing consequences for individual hospitals and the entire healthcare system. The Israeli healthcare leadership needs to plan and support expanding, equipping and staffing acute and chronic care units that are staffed by providers trained to care for such complex patients.

**Electronic supplementary material:**

The online version of this article (10.1186/s13584-019-0291-y) contains supplementary material, which is available to authorized users.

## Introduction

Mechanical ventilation is a life supporting modality often employed in the care of severely ill patients. The increasing use of mechanical ventilation parallels greater hospitalization in intensive care units and is attributed to an aging population, the advent of sophisticated surgical procedures, more aggressive medical, neurological and oncological treatments and improved treatment of critical illness [1–3]. Recent epidemiological research in the US revealed that about 310 persons per 100,000 adult population undergo invasive ventilation for nonsurgical indications [2]. This situation has led to increased healthcare expenditures [4, 5]. In the United States daily intensive care unit (ICU) costs are three to five times the costs of regular ward care [6, 7]. A portion of these higher costs is attributable to the equipment, supply and personnel expenditures required to care for ventilated patients [8]. However, mechanically ventilated patients are not only found in ICUs but also in intermediate care units, ventilator weaning units and patient wards [9–11]. In Israel, many seriously ill patients are mechanically ventilated in internal medicine wards due to a shortage of ICU beds [10, 12–14].

In an era of increasing awareness of healthcare costs it is important to be cognizant of the patterns of in-patient mechanical ventilator utilization not only within ICUs but also in other areas of the hospital. This study explores the prevalence patterns of mechanical ventilation using a dataset collected over a 20-year period (1997–2016) in a two-hospital Israeli medical system that includes a tertiary care medical center and a community hospital. We examined hospital-wide ventilator use and patterns of ventilation in various areas of the hospital ranging from the surgical ICU to the internal medicine floors. The aim was to determine patterns of ventilator use (e.g. seasonality, weekday vs. weekend).. Another goal was to provide useful information to healthcare leaders about budgeting, staffing, equipment purchasing and materials management. Among the hypotheses tested was that over 20 years there have been substantial increases in the prevalence of mechanical ventilation. Another hypothesis examined was that although the overall pattern of mechanical ventilator utilization is seasonal, this pattern is due to internal medicine and not surgical activity.

## Materials and methods

Data were assembled from an administrative database of the Hadassah-Hebrew University Medical Center. This two-hospital system includes a 900-bed tertiary care university hospital which serves as the major referral center and regional level 1 trauma center for a metropolitan area of over 1 million inhabitants. The other hospital is a 300-bed community hospital. The dataset includes the routine daily ventilator (both invasive and non-invasive) census collected at 8:00 AM over a 20-year period (1997 through 2016) by the Respiratory Care Service. The data were collected separately for each hospital unit that cared for ventilated patients. Patients not accommodated in medical intensive care units (MICU) are routinely ventilated on the internal medicine floors. However, surgical and pediatric patients are rarely ventilated outside an ICU.

Additionally, a detailed “snapshot” using data collected by the Respiratory Care Service of the age and duration-of-ventilation of individual patients on the internal medicine wards over 4 months in 2015 was analyzed.

### Data analysis

Data were entered onto Excel® spreadsheets (Microsoft Inc., Redmont, WA) where descriptive statistical analysis was performed. Further analysis was performed with Systat 12 (Systat, San Jose, CA) Continuous variables with normal distributions are reported as mean ± standard deviations, those with non-normal distributions are reported as medians. Inter and intra-group comparisons were performed with analysis of variance and Tukey post-hoc tests. Best-fit regression analysis examined the association of ventilator-days patterns with time and was also used to compare ventilation patterns between various units. Statistical significance was inferred at *p* < 0.05.

Mechanical ventilator prevalence data from each hospital and their individual units, including general (GICU), medical (MICU), pediatric, neonatal, cardiothoracic surgery (CSICU) intensive care units and internal medicine wards were subjected to time-series and frequency domain analysis. The latter used Fourier transformation, while the former used time-series analysis to examine the internal structure of each data-file seeking autocorrelation, short and long-term trends, seasonality and intra-week variation. Winter was defined as December to March.

This study was approved by the Institutional Review Board. Informed Consent was waived due to the retrospective nature of the study.

## Results

**Fig. 1:**
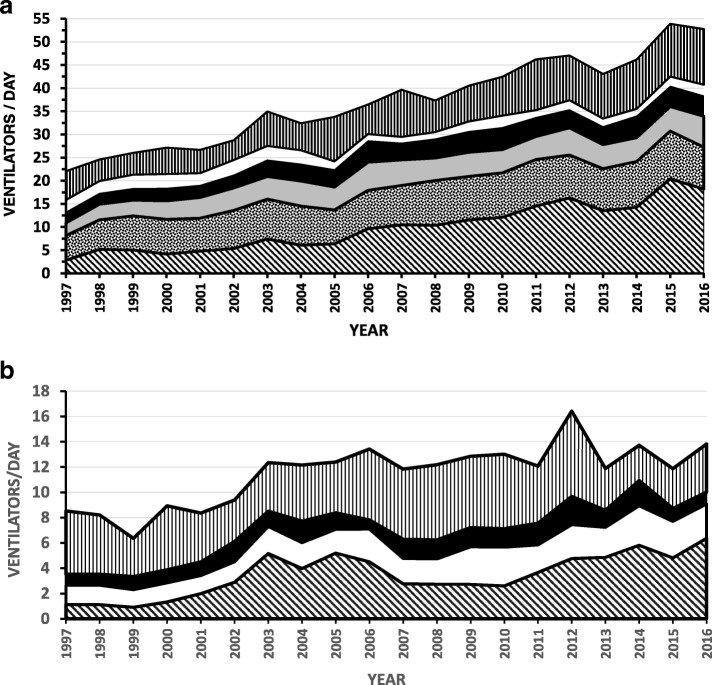
**a** University hospital: Over 20 years the average number of ventilated patients/day doubled mainly due to more patients ventilated on internal medicine wards (cross-hatch). There were much smaller increases in the general (speckled), medical (gray) and pediatric (black) ICUs, with no increases in the cardiothoracic surgical ICU (white). There were increases in other areas (vertical hatching) due mainly to increases in the emergency department and neurology wards. **b** Community hospital: Over 20 years the average number of ventilated patients/day increased over the initial 10 years and then remained essentially static. The increase was occasioned by more patients ventilated on internal medical wards (cross-hatch) and some increases in the medical/cardiac ICU (white) and neonatal ICU (vertical hatching). There were no changes in the surgical ICU (black). The decrease in the neonatal ICU during the last three years was due to a neonatal ICU opening at the university hospital

**Fig. 2:**
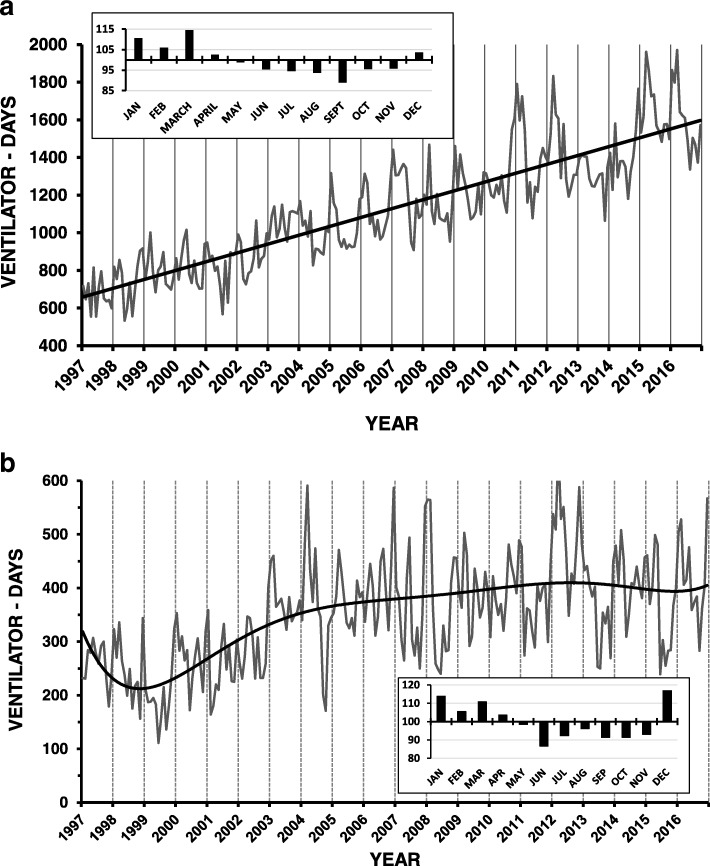
**a** University hospital: There was a linear increase in the number of monthly ventilator days over the twenty-year study period. Vertical gridlines indicate January of each year. The pattern was generally seasonal, especially during the latter half of the period, with peak ventilator use occurring during the winter. This was further demonstrated by the seasonality analysis (insert). **b** Community hospital: The total monthly ventilator days increased over the first ten-year period but remained static over the subsequent 10 years. Vertical gridlines indicate January of each year. The pattern was often seasonal, with peak ventilator use occurring during the winter as further demonstrated by the seasonality analysis (insert)

Over the 20-year period of 7305 days there were 294,456 and 92,208 ventilator-days at the university and community hospitals, respectively. Over two decades the total ventilator-days in the two hospitals increased from 11,164 (31 patients/day) in 1997 to 24,317 (67 patients/day) in 2016 mainly due to more patients ventilated on internal medicine wards (1997: 4 patients/day; 2016: 24 patients/day). The magnitude of the increase in ventilated patients was greater than the 40% increase in Jerusalem’s population over the 20-year period and also greater than the 55% increase in the over 65 years old population (1999: 57,300 people {7.7% of the population}; 2015: 88,000 people {8.4% of the population}) in the Jerusalem metropolitan area. Over the 20-year period the number of patients admitted to the university hospital increased 51% (1997: 51,243; 2016: 78,532) and the number admitted to the community hospital increased 31% (1997: 26,106; 2016: 34,258). In the university hospital, there were increases in the overall (1997: 22 patients/day; 2016: 53 patients/day) as well as internal medicine ward ventilator-days (1997: 3 patients/day; 2016: 18 patients per day). The latter increase was accompanied by the addition of two internal medicine wards (36 beds in 2000 and 15 beds in 2016) to the two wards (36 beds each) existing at the beginning of the study (Fig. 1). Each of these increases reduced the ventilator burden in the other internal medicine wards (for example, 1999: 26 ventilator-days per internal medicine ward bed per year; 2000: 14 ventilator-days per internal medicine ward bed per year). The increase in the overall number of monthly ventilator days in the university hospital was linear over the twenty-year study period (y = 3.92x + 655.54, r2 = 0.769). The linear regression equation for the first 10 years (y = 3.69x + 665.07, r2 = 0.51) predicted the increase over the subsequent 10-years (regression equation over last 10 years: (y = 3.85x + 671.51, r2 = 0.42) (Fig. 2).

There were increases in overall ventilator-days at the community hospital (1997: 9 patients/day; 2016: 14 patients/day) that plateaued after 10 years (best fit regression equation: y = 2E-10 × 6 - 1E-07 × 5 + 4E-05 × 4–0.0059 × 3 + 0.4421 × 2–12.653x + 330.97, r^2^ = 0.41), likely because of a saturation phenomenon due to lack of increases in internal medicine ward beds during the study period (two wards, total 72 beds) (Figs. 1b and 2b).

**Fig. 3:**
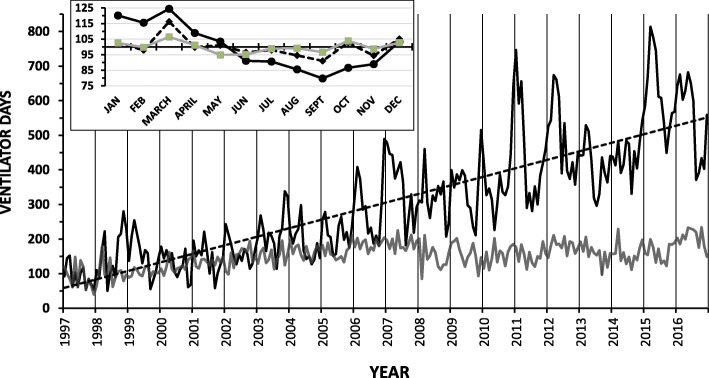
There was a marked linear (y = 2.06x + 56.80; r^2^ = 0.71) increase in mechanical ventilation prevalence over the 20-year period in the university hospital medical wards (black line), but not MICU (gray line). Vertical gridlines show January of each year. Most years there was a seasonal pattern on the medical wards, with peak ventilator use during the winter. Seasonality analysis (insert) shows winter peaks in the medical wards (black line) but not the MICU (dotted line) or GICU (gray line)

The monthly data from each of the two hospitals showed seasonal variation with the peak prevalence during the winter (Fig. 2b). There were seasonal variations in the internal medicine wards but not in the ICUs of the two hospitals (Fig. 3).

The increase in ventilator-days in the university hospital was mainly influenced by substantial ventilator-day increases in the internal medicine wards (r^2^ = 0.88, *p* < 0.0001) and to a much lesser degree by the GICU (r^2^ = 0.48, *p* < 0.001) and MICU (r^2^ = 0.40, *p* < 0.001) (Fig. 3). Other units showed smaller increases (pediatric intensive care units) or even small reductions (CSICU) in ventilator activity (Fig. 1). The frequency domain of the internal medicine wards had a major peak at a single frequency, while the pattern in the SICU was more heterogeneous.

**Fig. 4:**
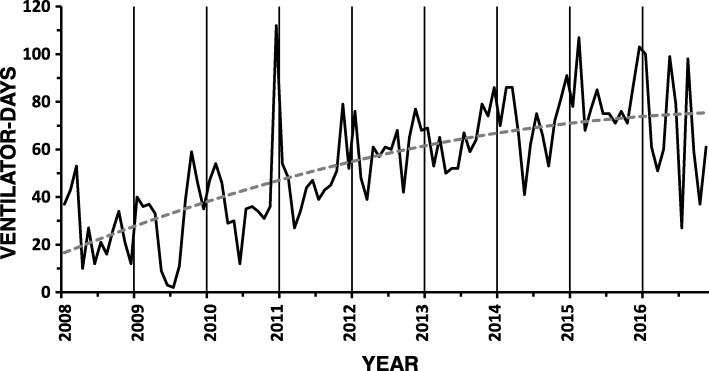
Monthly ventilator-days in the university hospital’s emergency department increased over the last 9 years of the study as displayed by the gray dashed regression line (y = − 0.01 × ^2^ + 1.02x + 15.62; r^2^ = 0.53). The vertical gridlines that indicate the January of each year illustrate that seasonal peaks occurred frequently during winter

Other units also showed distinctive patterns. Over the last 9 years of the study there was a steady increase in activity plus a seasonal pattern in the emergency department of the university hospital reflecting the patterns observed in the medical wards (Fig. 4). In the CSICU there was an intra-week pattern that reflected that cardiothoracic surgeons did not operate electively on Wednesdays and weekends (in Israel, Friday and Saturday) leading to significantly fewer ventilated patients on Thursday, Saturday and Sunday mornings. In the GICU the pattern reflected the increased trauma admissions during the weekend (Additional file 1: Table S1).

**Fig. 5:**
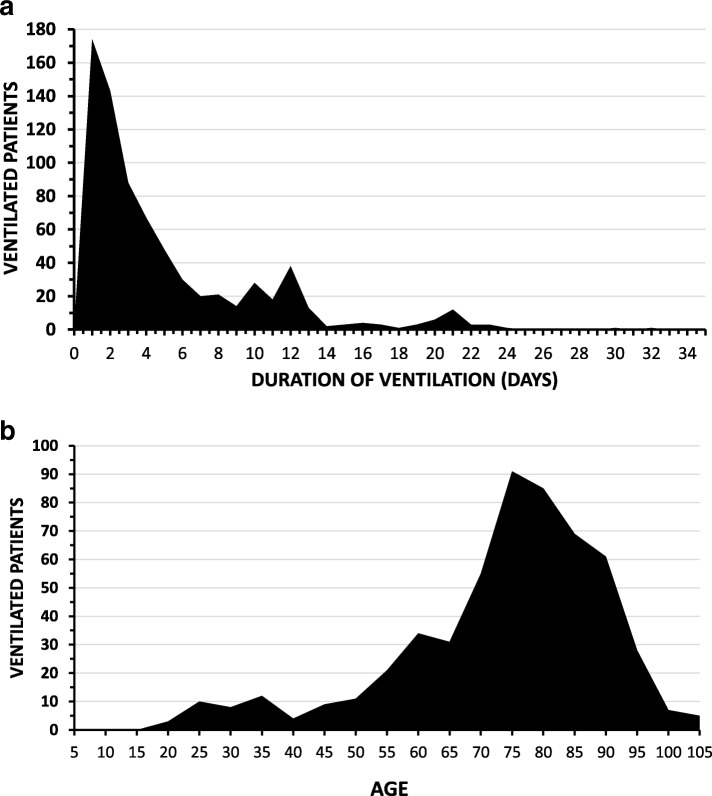
**a** The frequency distribution of the duration-of-ventilation of 745 patients ventilated on the university hospital’s internal medicine wards is rightward skewed. **b** Displayed is the frequency distribution of the ages of 745 patients ventilated on the internal medicine wards of the university hospital

The snapshot of individual patients (*n* = 745) ventilated on the internal medicine wards of the university hospital over 4 months in 2015 showed a duration-of-ventilation frequency distribution (Fig. 5) skewed to the right (mean duration - 5.1 ± 5.1 (SD) days, median – 3 days, mode – 1 day); 175 of the 745 (23.5%) patients were ventilated for greater than a week. The mean age was 71.5 ± 16.6 years (median 75 years) (Fig. 5b).

## Discussion

This study points out two important aspects of contemporary respiratory care: Increasing use of mechanical ventilation and various unit-specific patterns of mechanical ventilation. The major finding was that over the past two decades there was more than a doubling of ventilator-days in a two-hospital system located in Jerusalem mainly due to more patients ventilated in internal medicine wards. Although there were increases in other hospital areas they did not approach the magnitude of the increases seen in the internal medicine wards. These observations are consistent with those of other investigators who pointed out that the success of ICUs has vastly enhanced the ability and technology to support vital body functions, especially respiration [1–3]. As a result more and more patients are mechanically ventilated during their hospitalization with their numbers expected to rise in the future [15]. In the early 2000’s prolonged acute mechanical ventilation (≥96 h) in the United States increased at an annualized rate of 5.5% while the population increased by only 1% [16]. In the United States during 2005, 6,469,674 patients were hospitalized in six states with 180,326 (2.8%) receiving invasive mechanical ventilation. A total of 44.6% of the ventilated patients had at least one major co-morbidity; the most common being diabetes (13.2%) and pulmonary disease (13.2%). In-hospital mortality was 34.5% with only 30.8% of patients discharged home [4]. In Israel there are insufficient ICU beds causing many patients, especially elderly ones, to receive mechanical ventilation on internal medicine and neurology wards. This was demonstrated by the present study where the median age of patients ventilated on internal medicine wards was 75 years.

The hospital-wide ventilation patterns were the weighted sum of the patterns of individual patient units. The most apparent pattern was a seasonal one, with the peak prevalence during the winter due to seasonal patterns on the internal medical wards. This seasonal pattern is not surprising, given the greater incidence of pneumonia, influenza and chronic obstructive pulmonary disease (COPD) exacerbations during the winter [17–20]. In Wales, pneumococcal disease displayed a marked preponderance during winter [21]. Peaks also occur at other times of the year, with peak asthma exacerbations in Canadian children occurring during early September, correlating directly with the start of the school year [17], although others showed peak ICU asthma admissions during winter [22]. Therefore, on internal medicine and pediatric services seasonal variation among respiratory ailments typically occurs. We observed such a seasonal pattern in the adult emergency department, where ventilated patients often remained for extended periods while awaiting ICU or internal medicine ward beds. This situation contributes to the overload seen in Israeli emergency departments during winter [23].

Seasonal variation was not found in the GICU, which treated mainly surgical patients, nor in the CSICU or Neurosurgical ICU. This confirms the hypothesis that surgical ICUs do not contribute to the overall seasonality of mechanical ventilator use. This lack of seasonality can be explained by surgical illnesses, such as abdominal catastrophes and acute cardiac syndrome not being seasonal. However, trauma is often a seasonal occurrence, with peak incidence during summer [24–26]. A study of neurotrauma in Israel reported spring and summer peaks [27]. However, lack of seasonality in GICU mechanical ventilator use might be explained by a summer decrease in elective surgery requiring postoperative ICU care offsetting the increase in trauma [28]. The former decrease is attributable to surgeons, anesthesiologists and operating room nurses taking summer vacations thus decreasing elective surgical volume [28]. This complex pattern was demonstrated by the multi-peaked GICU frequency domain, unlike the singular-peaked pattern seen in the medical wards. Over the 20-year study there were small increases in GICU ventilator-days, despite a 60% increase in surgical volume and a 48% increase in GICU daily occupancy (C. Weissman, personnel communication). The introduction of advanced anesthetic and surgical techniques, including minimally invasive surgery, endovascular stents and angiographic control of traumatic hemorrhage have reduced postoperative mechanical ventilation, but not ICU admission. There was a small, but significantly greater number of ventilated GICU patients on Sunday than on Wednesday morning, likely reflecting a greater weekend trauma admission rate [29, 30]. The CSICU had a within-week pattern consistent with the weekdays that elective operations were performed. There were also fewer ventilated patients during April and September/October consistent with Israeli holiday periods and reduced elective surgery. A similar reduction in elective surgery coinciding with holidays was also observed with hip and knee replacement surgery in Canada [31, 32].

### Implications for hospitals

- Analyzing both overall and unit-specific prevalence patterns helps plan for sufficient and adequately equipped and staffed locations for safe mechanical ventilation.
- Areas with seasonal patterns, like internal medicine wards, require more nursing and respiratory care staff during the winter, while locations without seasonality require stable staffing throughout the year. This eases the burden on respiratory care departments when staff wish to take summer vacations [33].
- Most hospital monthly supply budgets are the same throughout the year. However, in units with marked seasonal variation, budgets for respiratory care and other supplies should have a seasonal pattern to prevent shortfalls during the winter [34, 35].

### Implications for the healthcare system

Ideally, hospitalized acutely ill mechanically ventilated patients should be cared for in settings, such as intensive and intermediate care units, where nurse:patient ratios are high (1:1 to 1:4) and medical and nursing staffs specially trained to care for complex respiratory problems, as well as in weaning such patients from their ventilators [36]. Optimally, patients unable to wean from their ventilators should be directly transferred either to specialized weaning units or to outside facilities that provide chronic ventilator care for further evaluation, weaning attempts and if weaning is unsuccessful, prolonged mechanical ventilation [37].

Israel has insufficient ICU beds causing many patients to receive mechanical ventilation on internal medicine, geriatric and neurology wards [38]. Izhakian and Buchs [39] found a 72% in-hospital mortality rate among 437 ventilated patients (median age 83 years) admitted directly from the emergency department to the medical wards of an Israeli hospital. This mortality rate was similar to reports from other Israeli medical wards. Studies from Israel, Britain and Hong Kong showed better survival of patients admitted directly to ICUs or transferred to an ICU shortly after starting mechanical ventilation, than those cared for exclusively on medicine wards [10, 13, 40–43]. However, this statement must be qualified since patients admitted to the ICU often having better functional status than those admitted to the wards [39]. In fact, some Israeli patients ventilated on medical or other wards were never presented for admission to the ICU staff.

### The challenge

Increases in mechanical ventilation challenges the healthcare system taxing hospital budgets, personnel, infrastructure and operations [9].

- Capital Expenditures: Purchasing mechanical ventilators and equipping wards with infrastructure (medical gases, physiologic monitors, centralized alarm systems) to support safe ventilation.
- Operating Costs: More respiratory supplies plus employing added respiratory and biomedical engineering technicians tasked with maintaining mechanical ventilators.
- Physicians and Nurses: Occupancy rates during winter routinely reach 105–110% of capacity in Israeli internal medicine wards, heavily burdening nurses and physicians since among the added patients, are high acuity ventilated ones [44]. These latter patients require both higher nurse:patient ratios and nurses and physicians with enhanced skill sets. However, budgetary limitations plus a nationwide nursing shortage often do not permit adequate staff expansion. This likely retards the ability to optimally care for these seriously ill patients likely leading to extended lengths-of-stay and reduced success with weaning from mechanical ventilation. In one report such patients had a one month post-discharge mortality of 79% [14].

### Possible solutions

Israeli healthcare expenditures are increasing due to an expanding and aging population coupled with new expensive and effective diagnostic and treatment modalities. Simultaneously, healthcare and hospital leaders must find solutions to adequately care for the increasing number of mechanically ventilated internal medicine and neurology patients, many of whom are elderly and suffer from multiple underlying chronic ailments.

- Adding ICU beds only addresses part of the problem given the many ventilated patients and greater expenses involved in building and operating ICU beds as opposed to intermediate care or ward beds.
- Higher per-diem reimbursement for mechanically ventilated patients. Although, over the 20-year period per-diem reimbursement has increased due to inflation and wage increases, no special reimbursement rate for non-ICU mechanically ventilated patients has been established. This lack of differential payments for mechanically ventilated patients has resulted in a situation where hospital revenues do not increase in accord with greater patient acuity and these patients’ increased personnel and equipment needs. This is part of a broader problem in Israel’s hospital reimbursement system where there are differential payments for some surgical admissions, which are generally reimbursed as Procedure Related Groups while there are no differential payments for internal medical admissions [45, 46].
- Increasing nurse staffing and improving knowledge and skills to better care for mechanically ventilated patients outside ICUs.
- Introducing bachelor degree level respiratory therapists to aid in caring and weaning mechanically ventilated patients. In the United States and Canada such licensed practitioners enhance the care of respiratory patients by relieving nurses of many technical and clinical respiratory care tasks plus providing specialized expertise in liberating patients from mechanical ventilation [47, 48].
- Physicians: The burden of caring for much of the increase in ventilated patients falls on internal and emergency medicine physicians, especially residents. However, often these physicians do not have specialized training in the technical and clinical aspects of mechanical ventilation [49, 50]. Also, most internal medicine residents plan to subspecialize in disciplines that do not care for such patients [51]. Whether internal medicine residency programs should include 3–4 months of required ICU training to improve residents’ skills could be considered. Another, possibility is training more critical care medicine specialists to consult on difficult to wean ventilated patients.
- Expand existing and add skilled nursing facilities capable of caring for chronically ventilated patients: In the current study, a quarter of ventilated internal medicine ward patients were ventilated for > 7 days. Many such patients are candidates for transfer to skilled nursing facilities but existing Israeli facilities are unable to meet demand resulting in chronically ventilated patients remaining in acute care hospitals for extended periods [52].
- The ethical issues of end-of-life care, advanced directives and living wills are crucial for the Israeli healthcare system. Among the reasons for publishing the present data was to spur socio-ethical research on mechanical ventilation and other life-supporting (extending) modalities, especially among patient populations ventilated on internal medicine, geriatric and neurology wards. Yet, this is a sensitive issue in Israel with its varied populations and religious beliefs.

### Strengths and limitations

This study’s major strength is that it analyzes in-patient mechanical ventilation using methods borrowed from econometrics, a discipline that specializes in exploring trends. Another strength was a dataset spanning 20 years that permitted us to examine both short and long-term trends. A limitation is that it included data from only two hospitals. However, research from other Israeli hospitals reported similar trends but over much shorter time frames [39, 37].

## Conclusions

Detailed analysis of the long-term trends of mechanical ventilation in a two-hospital system provided insight into a major issue facing Israeli healthcare. Increased prevalence of mechanical ventilation is a marker of the increasing age and illness severity of the hospitalized population, especially the denizens of internal medicine wards. This upward trend should continue as the proportion of the population over 65 years increases from 10 to 15% by 2035 [53]. Therefore, the healthcare leadership must plan to care for such patients, specifically, to provide financial support to adequately expand, equip and staff acute and chronic care facilities staffed by providers trained to care for such complex patients. This program should be supported by continuous data collection and statistical analysis similar to that performed in this study. Among other possible initiatives are higher per-diem hospital reimbursements for ventilated patients, training bachelor degree level respiratory therapists, improved training of nurses and house officers and establishing more dedicated weaning units in acute care hospitals and/or skilled nursing facilities. Moreover, the subject of advanced directives, living wills and end-of-life care should be addressed [54].

## Additional file

Additional file 1: **Table S1.** Weekly patterns of mechanical ventilation in the General ICU and the Cardiothoracic Surgical ICU over 20 years. (DOCX 13 kb)

## References

[1] Stefan MS, Shieh MS, Pekow PS, Hill N, Rothberg MB, Lindenauer PK (2015). Trends in mechanical ventilation among patients hospitalized with acute exacerbations of COPD in the United States, 2001 to 2011. Chest.

[2] Mehta AB, Syeda SN, Wiener RS, Walkey AJ (2015). Epidemiological trends in invasive mechanical ventilation in the United States: A population-based study. J Crit Care..

[3] Needham DM, Bronskill SE, Sibbald WJ, Pronovost PJ, Laupacis A (2004). Mechanical ventilation in Ontario, 1992–2000: incidence, survival, and hospital bed utilization of noncardiac surgery adult patients. Crit Care Med..

[4] Wunsch H, Linde-Zwirble WT, Angus DC, Hartman ME, Milbrandt EB, Kahn JM (2010). The epidemiology of mechanical ventilation use in the United States. Crit Care Med..

[5] Zilberberg MD, Luippold RS, Sulsky S, Shorr AF (2008). Prolonged acute mechanical, and mortality in the United States ventilation, hospital resource utilization. Crit Care Med..

[6] Dasta JF, McLaughlin TP, Mody SH, Piech CT (2005). Daily cost of an intensive care unit day: the contribution of mechanical ventilation. Crit Care Med.

[7] Kramer AA, Dasta JF, Kane-Gill SL (2017). The impact of mortality on total costs within the ICU. Crit Care Med.

[8] Jacobs P, Edbrooke D, Hibbert C (2001). Descriptive patient data as an explanation for the variation in average daily costs in intensive care. Anaesthesia.

[9] Latriano B, McCauley P, Astiz ME, Greenbaum D, Rackow EC (1996). Non-ICU care of hemodynamically stable mechanically ventilated patients. Chest.

[10] Simchen E, Sprung CL, Galai N, Zitser-Gurevich Y, Bar-Lavi Y, Gurman G, Klein M, Lev A, Levi L, Zveibil F, Mandel M, Mnatzaganian G (2004). Survival of critically ill patients hospitalized in and out of intensive care units under paucity of intensive care unit beds. Crit Care Med..

[11] Edwards JD, Rivanis C, Kun SS, Caughey AB, Keens TG (2011). Costs of hospitalized ventilator-dependent children: differences between a ventilator ward and intensive care unit. Pediatr Pulmonol.

[12] Lieberman D, Nachshon L, Miloslavsky O, Dvorkin V, Shimoni A, Zelinger J, Friger M, Lieberman D (2010). Elderly patients undergoing mechanical ventilation in and out of intensive care units: a comparative, prospective study of 579 ventilations. Crit Care..

[13] Hersch M, Sonnenblick M, Karlic A, Einav S, Sprung CL, Izbicki G (2007). Mechanical ventilation of patients hospitalized in medical wards vs the intensive care unit--an observational, comparative study. J Crit Care..

[14] Hersch M, Izbicki G, Dahan D, Breuer GS, Nesher G, Einav S (2012). Predictors of mortality of mechanically ventilated patients in internal medicine wards. J Crit Care..

[15] Donahoe MP (2012). Current venues of care and related costs for the chronically critically ill. Respir Care.

[16] Zilberberg MD, de Wit M, Pirone JR (2008). Growth in adult prolonged acute mechanical ventilation: implications for healthcare delivery. Crit Care Med..

[17] Johnston NW, Sears MR (2006). Asthma exacerbations 1: Epidemiology. Thorax.

[18] King JC, Ajao A, Lichenstein R, Magder LS (2014). Surge in hospitalizations associated with mechanical ventilator use during influenza outbreaks. Disaster Med Public Health Prep..

[19] Murdoch KM, Mitra B, Lambert S, Erbas B (2014). What is the seasonal distribution of community acquired pneumonia over time? A systematic review. Australas Emerg Nurs J..

[20] Moineddin R, Nie JX, Domb G, Leong AM, Upshur REG (2008). Seasonality of primary care utilization for respiratory diseases in Ontario: A time-series analysis. BMC Health Serv Res.

[21] Melegaro A, Edmunds WJ, Pebody R, Miller E (2006). George R: the current burden of pneumococcal disease in England and Wales. J Infections.

[22] Pendergaft TB, Stanford RH, Beasley R, Stempel DA, McGlaughlin T (2005). Seasonal variation in asthma-related hospital and intensive care admissions. J Asthma.

[23] Mudumbai SC, Barr J, Scott J, Mariano ER, Bertaccini E, Nguyen H, Memtsoudis SG, Cason B, Phibbs CS, Wagner T (2015). Invasive mechanical ventilation in California over 2000–2009: implications for emergency medicine. West J Emerg Med..

[24] Bhattacharyya T, Milham FH (2001). Relationship between weather and seasonal factors and trauma admission volume at a Level I trauma center. J Trauma.

[25] Rising WR, O'Daniel JA, Roberts CS (2006). Correlating weather an trauma admissions at a Level 1 trauma center. J Trauma.

[26] Parslow RC, Morris KP, Tasker RC (2005). Epidemiology of traumatic brain injury in children receiving intensive care n the UK. Arch Dis Child.

[27] Levi L, Linn S, Feinsod M, Revach M (1989). Neurotramatological survey in northern Israel. I. Annual and seasonal variations. Neuroepidemiology.

[28] Pettinger N (1999). Winter pressures. Lazy days of summer. Health Ser J.

[29] Soffer D, Zmora O, Klausner JB, Szold O, Givon A, Halpern P, Schulman CI, Peleg K (2006). Alcohol use among trauma victims admitted to a level I trauma center in Israel. Isr Med Assoc J..

[30] Jaffe DH, Savitsky B, Zaistev K, Hiss J, Peleg K (2009). Alcohol and driver fatalities in Israel: an examination of the current problem. Isr Med Assoc J..

[31] Ross EGU, Moineddin R, Crighton EJ, Mamdani M (2006). Seasonality of service provision in hip and knee surgery: a possible contributor to waiting times? A time series analysis. BMC Health Serv Res.

[32] Moore IC, Strom DP, Vargas LG, Thomson DJ (2008). Observations on surgical demand time series. Anesthesiology.

[33] Schmidt L, Nelson D (1996). A seasonal staffing model. J Nurs Adm.

[34] David C (1999). Maddox Budgeting for Not-for-Profit Organizations John Wiley and Sons.

[35] Andrew E, William Cleverley (2006). Cameron Essentials of Health Care Finance Jones & Bartlett Publishers.

[36] Kydonaki K, Huby G, Tocher J (2016). Understanding nurses' decision-making when managing weaning from mechanical ventilation: a study of novice and experienced critical care nurses in Scotland and Greece. J Clin Nurs..

[37] Kaykov E, Vigder C, Ben Nathan M (2014). Identifying predictors of successful weaning off prolonged mechanical ventilation among the elderly in an Israeli Respiratory Care Facility. Int J Caring Sci.

[38] Abramovitch A, Friedmann R, Zevin S, Munter G, Yinnon AM, Raveh-Brawer D (2017). Operating a monitoring unit in the geriatric department: effects on obutcomes. J Am Geriatr Soc..

[39] Izhakian S, Buchs AE (2015). Characterization of patients who were mechanically ventilated in general medical wards. IMAJ.

[40] Naser W, Schwartz N, Finkelstein R, Bisharat N (2016). Outcome of mechanically ventilated patients initially denied admission to an intensive care unit and subsequently admitted. Euro J Int Med.

[41] Tang WM, Tong CK, Yu WC (2012). Outcome of adult critically ill patients mechanically ventilated on general medical wards. Hong Kong Med J.

[42] Metcalfe MA, Slogett A, McPherson K (1997). Mortality among appropriately referred patients refused admission to intensive care units. Lancet..

[43] Frisho-Limo P, Gurman G, Schapira A, Porath A (1994). Rationing critical care—what happens to patients who are not admitted?. Theoretical Surgery..

[44] http://www.cbs.gov.il/publications17/yarhon0917/pdf/d1.pdf Accessed 22 Oct 2017.

[45] Ministry of Health Price List for Ambulatory and Hospitalization Services. https://www.health.gov.il/Subjects/Finance/Taarifon/Pages/PriceList.aspx. Accessed 7 Jan 2019.

[46] Greenberg RW, Waitzberg R, Perman R, Gamzu R (2015). Why and how did Israel adopt activity-based hospital payment? The Procedure-Related Group incremental reform. Health Policy.

[47] Merendino D, Wissing DR (2005). New roles for respiratory therapists: expanding the scope of practice. Respir Care Clin N Am..

[48] Radosevich MA, Wanta BT, Meyer TJ, Weber VW, Brown DR, Smischney NJ, Diedrich DA: Implementation of a goal-directed mechanical ventilation order set driven by respiratory therapists improves compliance with best practices for mechanical ventilation. J Intensive Care Med. 2017 :10.1177/0885066617746089. 10.1177/0885066617746089 29207907

[49] Wilcox SR, Strout TD, Schneider JI, Mitchell PM, Smith J, Lutfy-Clayton L, Marcolini EG, Aydin A, Seigel TA, Richards JB (2016). Academic emergency medicine physicians' knowledge of mechanical ventilation. West J Emerg Med..

[50] Wilcox SR, Seigel TA, Strout TD, Schneider JI, Mitchell PM, Marcolini EG, Cocchi MN, Smithline HA, Lutfy-Clayton L, Mullen M, Ilgen JS, Richards JB (2015). Emergency medicine residents' knowledge of mechanical ventilation. J Emerg Med..

[51] Cox CE, Carson SS, Ely EW, Govert JA, Garrett JM, Brower RG, Morris DG, Abraham E, Donnabella V, Spevetz A, Hall JB (2003). Effectiveness of medical resident education in mechanical ventilation. Am J Respir Crit Care Med..

[52] Cox CE, Carson SS (2012). Medical and economic implications of prolonged mechanical ventilation and expedited post-acute care. Semin Respir Crit Care Med..

[53] http://www.cbs.gov.il/reader/shnaton/templ_shnatone.html?num_] tab=st02_10&CYear=2017. (Accessed 18 Nov 2017).

[54] Fritch J, Petronio S, Helft PR, Torke A (2013). Making decisions for hospitalized older adults: ethical factors considered by family surrogates. J Clin Ethics.

