# Supplementary material for: Mechanical ventilation patterns and trends over 20 years in an Israeli hospital system: policy ramifications
Source: Isr J Health Policy Res. 2019 Feb 1;8:20. doi: 10.1186/s13584-019-0291-y (PMC6357444; doi:10.1186/s13584-019-0291-y)
Supplement: Supplementary file 1 — Table S1. Weekly patterns of mechanical ventilation in the General ICU and the Cardiothoracic Surgical ICU over 20 years. (DOCX 13 kb) [file 13584_2019_291_MOESM1_ESM.docx]

Additional file 1: Table S1. Weekly patterns of mechanical ventilation in the General ICU and the Cardiothoracic Surgical ICU over 20 years.

Day of the Week Cardiothoracic Surgery ICU§ General ICU

Sunday 1.92 ± 0.79 8.60 ± 1.23

Monday 2.77 ± 0.88* 8.54 ± 1.17

Tuesday 2.80 ± 0.90 8.41 ± 1.24

Wednesday 2.63 ± 0.94 8.40 ± 1.26****

Thursday 1.36 ± 0.32** 8.41 ± 1.26

Friday 2.48 ± 0.95 8.47 ± 1.35

Saturday 1.58 ± 0.35** * 8.52 ± 1.26

Values are ventilators per day ± standard deviation at 8:00 am (many cardiac surgical patients are extubated before 8:00 am)

§ The cardiothoracic surgery service does not perform elective surgery on Wednesdays.

*vs Sunday p<0.001 *** vs Friday p<0.001

** vs Wednesday p<0.001 ****vs Sunday p<0.02
